# Endobronchial Ultrasound Guided Transbronchial Needle Aspiration and Next Generation Sequencing Yields

**DOI:** 10.1007/s00408-024-00690-6

**Published:** 2024-04-30

**Authors:** Kristin N. Sheehan, Lara M. Khoury, Angela G. Niehaus, William I. Mariencheck, Katherine A. Gershner, Travis L. Dotson, Christina R. Bellinger

**Affiliations:** 1 Department of Pulmonary/Critical Care, Wake Forest University School of Medicine, Winston-Salem, NC 27157 USA; 2 Department of Internal Medicine, Wake Forest University School of Medicine, Winston-Salem, NC 27157 USA; 3 Department of Pathology, Wake Forest University School of Medicine, Winston-Salem, NC 27157 USA

**Keywords:** EBUS-TBNA, Bronchoscopy, Next generation sequencing, Molecular markers, Lung cancer

## Abstract

**Purpose:**

The use of endobronchial ultrasound (EBUS) is standard practice for lung cancer diagnosis and staging. Next generation sequencing (NGS) for detection of genetic alterations is recommended in advanced, non-squamous, non-small-cell lung cancer (NSCLC). Existing protocols for NGS testing are minimal and reported yields vary. This study aimed to determine the yield of EBUS samples obtained for NGS using a sampling protocol at our institution and assess predictive factors to form collection protocols.

**Methods:**

We reviewed EBUS bronchoscopies from 2016 to 2021 with non-squamous NSCLC diagnoses. For target lesions suspected to be malignant, the sampling protocol was: (a) two slides for on-site evaluation, (b) three to five fine needle aspirations rinsed into saline for immunohistochemical staining and in-house molecular markers, and (c) additional three to five rinses for NGS. Sufficiency for NGS processing was determined by the pathology department.

**Results:**

Two hundred and seventy-eight non-squamous NSCLC samples were obtained by EBUS (205 adenocarcinoma; 73 not otherwise specified). EBUS was performed under general anesthesia in 75.5% of cases. The overall sample adequacy for NGS testing was 57.5%. Higher adequacy rates were observed when protocol was adhered to 66.0% versus 37.2% (*p* < 0.001). There was no statistically significant difference based on the size of the lesion or location of the sample.

**Conclusion:**

When a protocol of three to five dedicated needle rinses for NGS was followed, we nearly doubled our sample adequacy rate for NSG as compared to standard care. Studies are needed to determine the ideal collection and processing modality to preserve tissue samples for genetic sequencing.

## Introduction

Endobronchial ultrasound (EBUS) is standard practice for the diagnosis and staging of lung cancers. Previous studies have shown that EBUS is highly accurate, cost effective, and can minimize diagnostic work up time [1–6]. EBUS is used to obtain tissue for molecular analysis which plays a vital role in targeted lung cancer treatment [3, 7]. Common mutations tested include epidermal growth factor receptor (EGFR), anaplastic lymphoma kinase (ALK), and c-ros oncogene 1 (ROS1), as well as many others [8]. The number of tested mutations continues to grow, further justifying the need for next generation sequencing (NGS) to identify these and other potentially targetable mutations.

NGS for detection of DNA and RNA alterations has been recommended for routine use in advanced, non-squamous, non-small-cell lung cancer, among other cancers [9]. NGS has been shown to have advantages, including higher sensitivity and accuracy, compared to more traditional approaches such as quantitative PCR and Sanger sequencing [10]. Existing literature on protocols for NGS testing is minimal and previously reported yields range from 40 to 95% [11, 12]. The objective of this study was to determine the yield of EBUS samples obtained for NGS at a single academic institution and assess predictive factors that can be used to form collection protocols.

## Methods

**Fig. 1:**
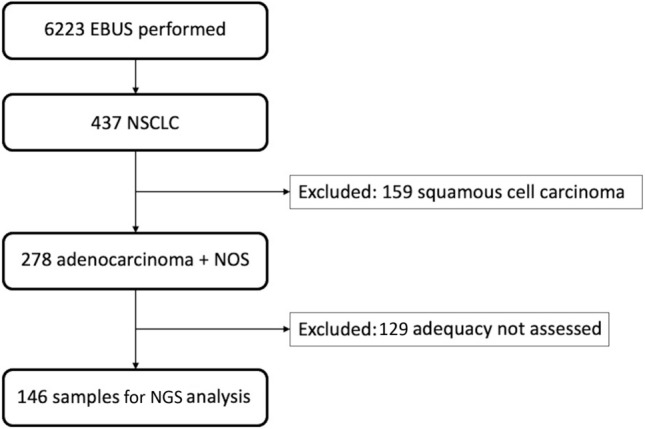
Consort diagram. *EBUS *endobronchial ultrasound; *NSCLC *non-small cell lung cancer; *NOS *not otherwise specified

We reviewed EBUS bronchoscopies performed at Atrium Health Wake Forest Baptist Medical Center (AHWFBMC), a regional academic tertiary care center, from January 2016 to December 2021 with cancer diagnoses. Only patients with primary non-small cell lung cancers (NSCLC), specifically adenocarcinoma or not otherwise specified (NOS) were included (Fig. 1).

Attending physicians were experienced bronchoscopists in advanced diagnostic modalities who performed or directly supervised all procedures [13–15]. EBUS was performed using an Olympus® BF-Convex Scope XBF-UC160 F-OL8 with a 21g needle or Pentax® convex scope EB-1970UK with a 21g cytology needle. Sedation was achieved with either general anesthesia or moderate sedation using fentanyl and midazolam as determined by the proceduralist. Demographics and procedural information was collected retrospectively. Masses were defined as lesions 3 cm or larger.

Our sampling protocol recommends a minimum of three fine needle aspirations (FNAs) from each EBUS target. Two dedicated needle passes from each target lesion are used to create four slides, two that are stained using the Diff-Quick method and immediately reviewed by an on-site cytotechnician for adequacy. The other two slides are placed into ethanol fixative for Papanicolai staining in the cytopreperatory lab. An additional third needle pass is rinsed directly into saline to create a cell block for processing by pathology. Once a suspected malignant target is identified, the bronchoscopist collects an additional three needle passes for dedicated rinses into saline for immunohistochemical stains and standard molecular genetic testing. If NSCLC is suspected, an additional three to five rinses are placed in a separate conical of saline and labeled for NGS testing. Data on total number of needle passes for each EBUS was obtained retrospectively from bronchoscopy procedure reports documented in the electronic health record (EHR).

EGFR, ALK, and ROS-1 are performed under a standard reflex pathology testing protocol. If preliminary pathology is consistent with adenocarcinoma, NOS, or poorly differentiated carcinoma, the samples are evaluated for ALK, ROS-1, and EGFR mutations without consulting the ordering physician or oncologist. Gene mutation testing is done either by fluorescent in situ hybridization (FISH) or DNA polymerase chain reaction sequencing at an offsite Clinical Laboratory Improvement Amendments-certified laboratory [15]. Per literature standards it is believed that greater than 100 tumor cells are needed for FISH and tumor content of 50–70% is required for mutational analysis [15].

At the time of this study, NGS testing was outsourced to Foundation Medicine for FoundationOne® companion diagnostic (CDx) testing and required Oncology request with patient consent [16]. Once a request for NGS reaches the pathology department, the histologic slides are reviewed by a pathologist (H&E stained slides) to assure adequate tumor remains for testing and for the selection of tumor blocks. FoundationOne®CDx requires a minimum of 20% tumor nuclei, with optimum 30% tumor nuclei [16]. Tumor content is calculated by the number of tumor cells divided by the total of nucleated cells [16]. All tissue is processed using standard fixation methods, 10% neutral-buffered formalin, to assure the preservation of nucleic acids [16]. Formalin fixed paraffin embedded tissue or 10 unstained slides, cut at 4 μm, and mounted on positively charged slides are accepted for testing [16]. Once Foundation Medicine receives the tissue, sequencing takes approximately 12–17 days. A finalized report is sent to medical records to be uploaded into the patient’s chart.

Descriptive statistics, means and standard deviation, median and range, or counts and percentages, were calculated to describe the patient, lesion, and procedural characteristics. Chi-square tests were used to compare the distribution of lesion and procedural characteristics for sufficient and insufficient samples for each of the mutations (EGFR, ALK, and ROS-1) and NGS testing. Results were considered significant when the *p* value was ≤ 0.05. The analysis for this paper was generated using SAS software, version 9.4 (SAS Institute Inc., Cary, NC).

## Results

### Patient and Bronchoscopy Characteristics

Between January 2016 and December 2021, a total of 6223 EBUS procedures were performed. Of these, 278 were non-squamous NSCLC samples. 54.3% were male and 56.1% of patients had stage IV disease. The mean patient age was 66.9 ± 10.6 years. Sampling location and lesion size are detailed in Table 1.

**Table 1:** Patient demographics and lesion characteristics

	All	Adeno	NOS
*N*	278	205	73
Age, mean (SD)	66.9 (10.6)	67.2 (11.1)	66.2 (9.0)
Gender			
Female	127 (45.7%)	97 (47.3%)	30 (41.1%)
Male	151 (54.3%)	108 (52.7%)	43 (58.9%)
Number of sites sampled, median (min; max)	2.0 (1.0; 8.0)	2.0 (1.0; 8.0)	2.0 (1.0; 7.0)
1–2	159 (57.2%)	117 (57.1%)	42 (57.5%)
3 or more	119 (42.8%)	88 (42.9%)	31 (42.5%)
Primary location			
Parenchymal nodule/mass	29 (10.4%)	22 (10.7%)	7 (9.6%)
High paratracheal (2R/2L)	1 (0.4%)	1 (0.5%)	0 (0.0%)
L paratracheal (4L)	14 (5.0%)	9 (4.4%)	5 (6.8%)
R paratracheal (4R)	67 (24.1%)	49 (23.9%)	18 (24.7%)
Left hilum (10L/11L)	35 (12.6%)	28 (13.7%)	7 (9.6%)
Right hilum (10R/11R)	54 (19.4%)	36 (17.6%)	18 (24.7%)
Subcarinal (7)	78 (28.1%)	60 (29.3%)	18 (24.7%)
Cancer stage			
Stage I	17 (6.1%)	14 (6.8%)	3 (4.1%)
Stage II	16 (5.8%)	11 (5.4%)	5 (6.8%)
Stage IIIA	45 (16.2%)	33 (16.1%)	12 (16.4%)
Stage IIIB	44 (15.8%)	34 (16.6%)	10 (13.7%)
Stage IV	156 (56.1%)	113 (55.1%)	43 (58.9%)

The most common primary sampling site was from subcarinal lymph nodes. The median number of sites sampled was 2, but ranged between 1 and 8, with greater than half falling within the 1–2 sites category as expected with the majority of patients being stage IIA or higher (79.9%).

Three quarters of the EBUS procedures were performed under general anesthesia (75.5%). The average procedure duration was 36.9 ± 13.0 min for moderate sedation and 51.6 ± 20.2 min for general anesthesia. There were 7 (2.5%) procedure related complications, which included hypoxia requiring admission (0.4%, *n* = 1), transient bronchospasm or desaturation (1.1%, *n* = 3), overall poor tolerance of the procedure (0.7%, *n *= 2), and other complications not specified (0.4%, *n* = 1). There were no reports of pneumothorax, hemoptysis, pneumonia, hypotension, arrhythmia, or death (Table 2).

**Table 2:** Procedural characteristics

General anesthesia (*n*, %)	210 (75.5%)
Procedure time, mean (SD)	51.6 (20.2)
Moderate sedation (*n*, %)	68 (24.4%)
Procedure time, mean (SD)	36.9 (13.0)
Complications (*n*, %)	
None	271 (97.5%)
Hypoxia requiring admission	1 (0.4%)
Poor overall tolerance of procedure	2 (0.7%)
Transient bronchospasm or desaturation	3 (1.1%)
Other	1 (0.4%)

### Pathology and Molecular Genetics

The details of types of cancer diagnoses and staging are listed in Table 1. 73.7% of cancers were diagnosed as adenocarcinoma (*n* = 205), while 26.3% were labeled NOS (*n* = 73).

**Fig. 2:**
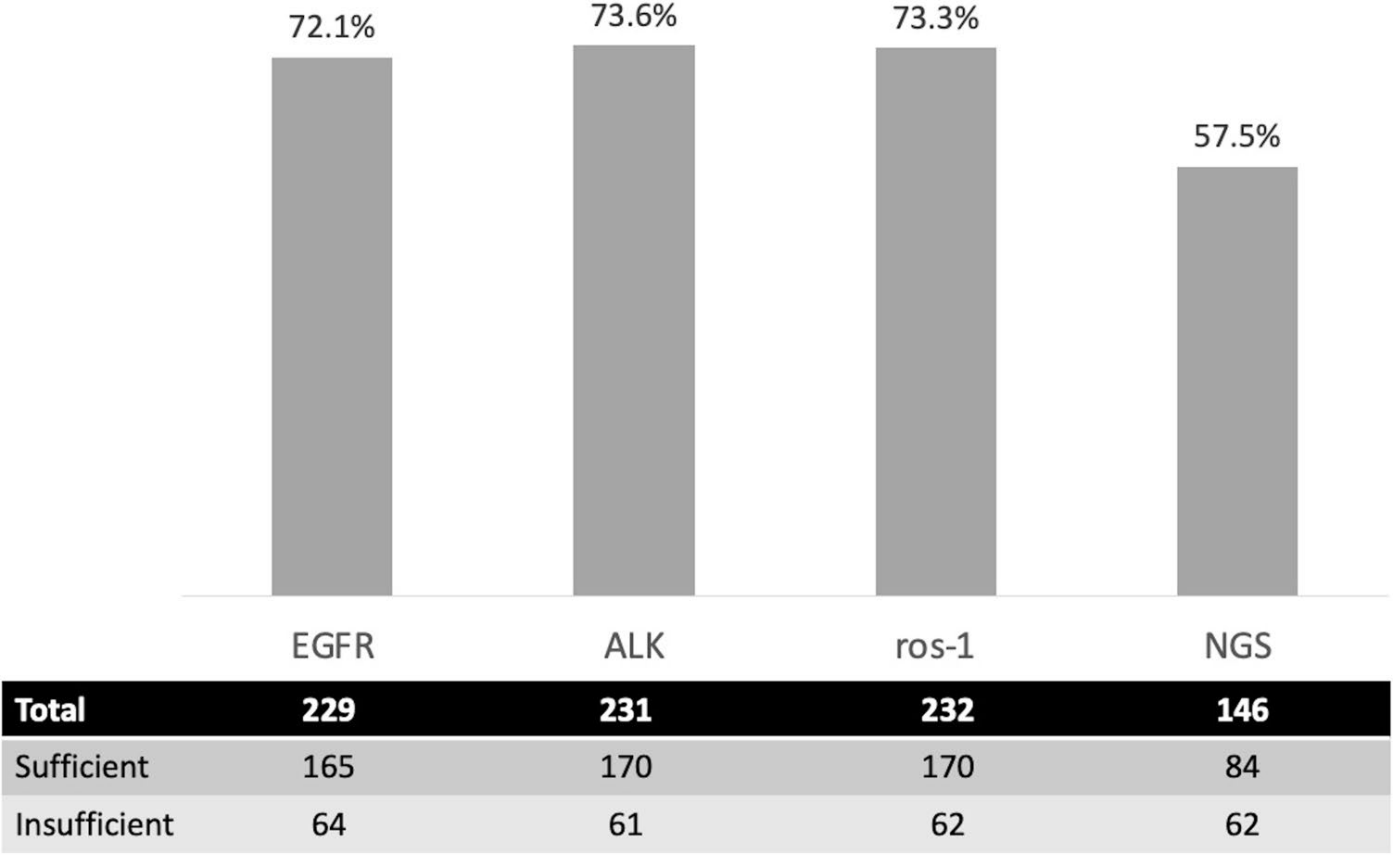
Sufficiency of samples for EGFR, ALK, ros-1, and NGS. Samples that were tested for EGFR, ALK, and ros-1 mutations as well as NSG, were evaluated by pathology to determine sufficiency of the sample to run molecular testing. 72.1% of samples were sufficient for EGFR, 73.6% for ALK, 73.3% for ros-1, and 57.5% for NGS

EGFR was tested in 229 samples and 72.1% (165) of these samples were sufficient for molecular testing (Fig. 2).

Among the adequate samples tested for EGFR, 15 samples (9.1%) were positive for the mutation. Tissue tested for EGFR was obtained from the mediastinal or hilar mass or nodule in 203 patients and from lymph nodes in 26 patients (Table 3).

**Table 3:** Molecular genetic sufficiency in adenocarcinoma, NOS, and poorly differentiated samples

	EGFR		*p*-value
	Insufficient	Sufficient	
*N*	64	165	
Primary location			0.7335
Mass/nodule	56 (87.5%)	147 (89.1%)	
Lymph node	8 (12.5%)	18 (10.9%)	
Size			0.0428
Number of aspirations			0.5731

	ALK		*p*-value
	Insufficient	Sufficient	
*N*	61	170	
Primary location			0.6826
Mass/nodule	55 (90.2%)	150 (88.2%)	
Lymph node	6 (9.8%)	20 (11.8%)	
Size			0.0115
Number of aspirations			0.2702

	ROS1		*p*-value
	Insufficient	Sufficient	
*N*	62	170	
Primary location			0.6556
Mass/nodule	56 (90.3%)	150 (88.2%)	
Lymph node	6 (9.7%)	20 (11.8%)	
Size			0.0106
Number of aspirations			0.2105

**Fig. 3:**
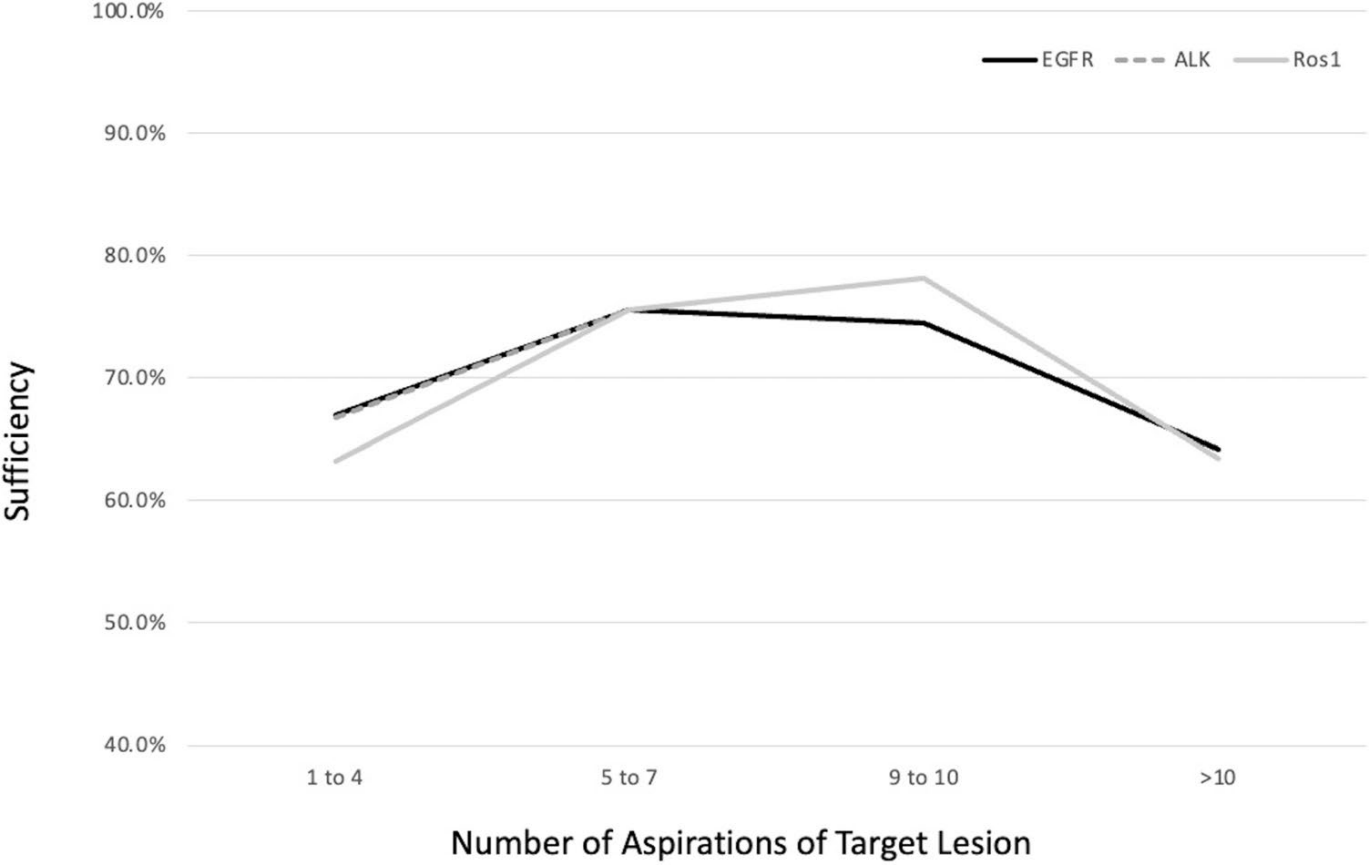
Molecular genetic sufficiency stratified by number of aspirations of target lesion. Tissue samples sufficient for molecular testing (EGFR, ALK, and ros-1) were stratified by the number of aspirations obtained from the target lesion with the highest yields obtained with 5–10 aspirations

Neither the location of sample, nor the number of aspirations had a significant effect on tissue sufficiency for testing (Fig. 3).

**Fig. 4:**
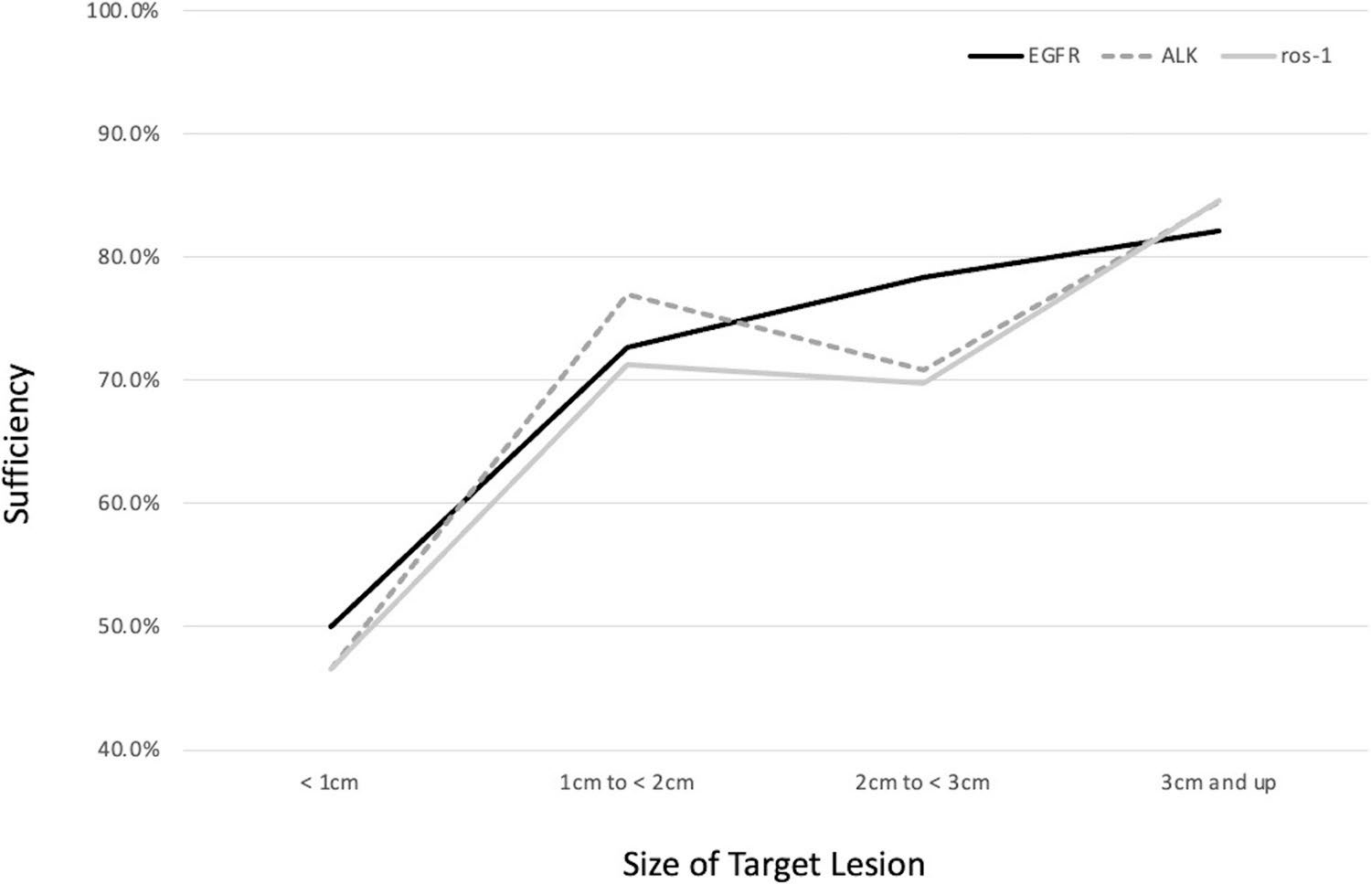
Molecular genetic sufficiency stratified by size of target lesion. Tissue samples sufficient for molecular testing (EGFR, ALK, and ros-1) were stratified by the size of the target lesion and showed a general trend toward increased sufficiency with larger lesions

However, the size of the lesion was significant, with those measuring 3 cm or greater having the highest yields (Fig. 4).

The presence of ALK mutations were tested in 231 samples. 73.6% (170) of samples were sufficient and seven of those were positive for the mutation (4.1%) (Fig. 2). Tissue tested for ALK was obtained from the mediastinal or hilar mass or nodule in 205 patients and from lymph nodes in 26 patients (Table 3). There was no significant difference in sample yields based on the location of sample or number of aspirations (Fig. 3). The size of the lesion was significant with those measuring 3 cm or greater having the highest yields (Fig. 4).

ROS-1 mutations were tested in 232 samples, of which 73.3% (170) samples were found to be sufficient and 4 of those were positive for the mutation (2.4%) (Fig. 2). The tissue tested for ROS-1 was obtained from the mediastinal or hilar mass or nodule in 206 patients and from lymph nodes in 26 patients (Table 3). There was no significant difference in sample yields based on the location of sample or number of aspirations (Fig. 3). The size of the lesion was significant with those measuring 3 cm or greater having the highest yields (Fig. 4).

The protocol of 3–5 dedicated passes was adhered to in 64.0% of cases. NGS was completed in 146 samples (Table 4).

**Table 4:** Next generation sequencing sufficiency in adenocarcinoma and poorly differentiated samples

	Next generation sequencing			*p*-value
	Not tested	Insufficient	Sufficient	
*N*	129	62	84	
Primary location				0.8051
Parenchymal nodule/mass	9 (7.0%)	9 (14.5%)	11 (13.1%)	
Lymph node	120 (93.0%)	53 (85.5%)	73 (86.9%)	
Paratracheal [high paratracheal (2R/2L), L paratracheal (4L), R paratracheal (4R)]	33 (25.6%)	19 (30.6%)	28 (33.3%)	
Hilum [left hilum (10L/11L), right hilum (10R/11R)]	45 (34.9%)	21 (33.9%)	23 (27.4%)	
Subcarinal (7)	42 (32.6%)	13 (21.0%)	22 (26.2%)	
Size				0.0856
< 1 cm	7 (5.4%)	8 (12.9%)	3 (3.6%)	
1 to < 2 cm	29 (22.5%)	12 (19.4%)	19 (22.6%)	
2 to < 3 cm	41 (31.8%)	23 (37.1%)	27 (32.1%)	
≥ 3 cm	47 (36.4%)	14 (22.6%)	30 (35.7%)	
Unknown	5	5	5	
Cancer stage				0.0615
Stage I	8 (6.2%)	7 (11.3%)	2 (2.4%)	
Stage II	10 (7.8%)	2 (3.2%)	4 (4.8%)	
Stage IIIA	21 (16.3%)	14 (22.6%)	10 (11.9%)	
Stage IIIB	22 (17.1%)	8 (12.9%)	12 (14.3%)	
Stage IV	68 (52.7%)	31 (50.0%)	56 (66.7%)	

**Fig. 5:**
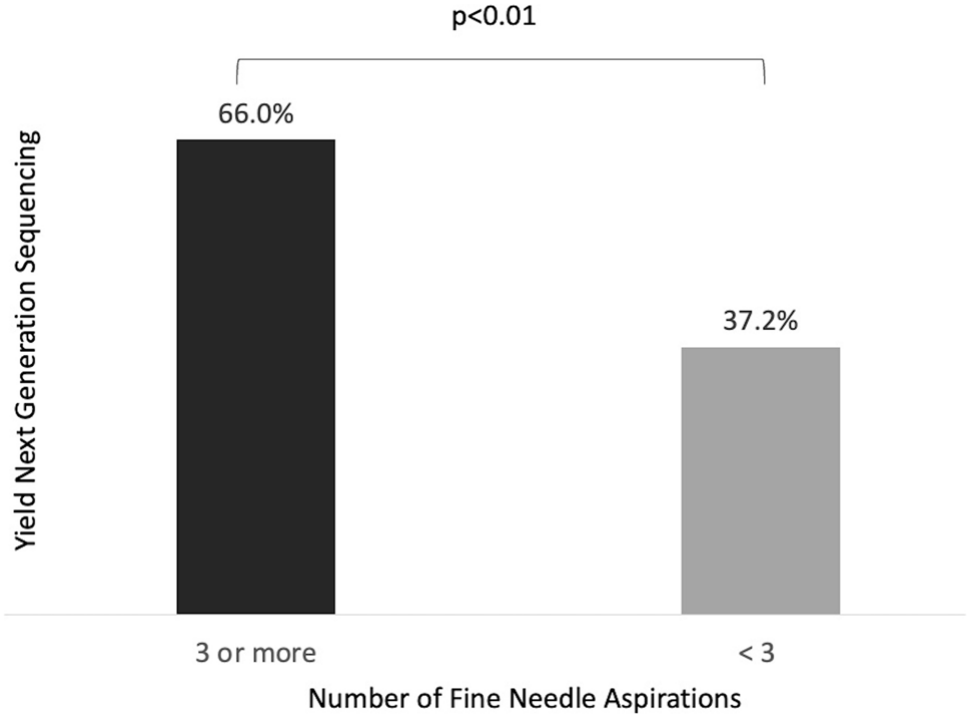
Next generation sequencing yields based on protocol adherence (≥ 3 aspirations). Samples tested for next generation sequencing were stratified by number of aspirations taken from the target lesion. Yields were higher when three or more aspirations were obtained compared to less than three aspirations (66% vs 37.2%)

The overall yield for NGS was 57.5% (Fig. 1). When stratified based on lesion size, adequacy was 52.4% for lesions < 2 cm, 54.0% for 2 to < 3 cm, and 68.2% for samples ≥ 3 cm, none of which were statistically significant (Fig. 3). There was no statistically significant difference in yields based on the primary sampling location or cancer stage. Higher yields were observed when the protocol of 3–5 dedicated passes was adhered to: 66.0% versus 37.2% (*P* = 0.0013) (Fig. 5).

## Conclusion

Because of the success of targeted cell therapies for treating non-small cell lung cancers, the need for accurate diagnostic capabilities is paramount. EBUS-TBNA is the standard, minimally invasive method to obtain diagnostic samples, and can be used to obtain material for next generation sequencing. Adequate collection of samples is essential to guide treatment decisions based on driver mutation status of ALK, EGFR, and ROS-1. However, there are no definitive guidelines on procedure protocols needed to obtain adequate samples for NGS testing.

In our study we analyzed samples from 278 patients with non-squamous non-small cell lung cancers, of which, 146 underwent NGS using tissue samples. When the protocol was adhered to, higher NGS yields were obtained, 66.0% versus 37.2% (*p*  < 0.01); however it was only followed 64.0% of the time. Despite lower than expected adherence to the protocol, we found that samples were sufficient for EGFR, ALK, and ROS-1 testing > 70% of the time. This number fell to 57.5% for NGS testing. We identified the only consistent predicting factor for sufficiency to be larger lesion size for EGFR, ALK, and ROS-1 testing, and number of aspirations for NGS testing. Reasons for differences in significant factors between NGS testing and individual mutation testing is unclear but may be due to laboratory processing requirements or the need to obtain more tissue to run multiple genetic tests with NGS rather than just one specific mutation. NGS testing was requested across all stages of lung cancer but was not found to be associated with NGS sufficiency. Although the oncologist’s reason for ordering NGS in individual patients was not collected in our data set, early-stage ordering is a reflection of many factors such as planned combined use of immunotherapy and chemotherapy, anticipation of future disease progression, and the need for treatment options in patients who are not surgical candidates. While NGS is standard in advanced NSCLC, recent trials have suggested variable benefit in early-stage disease as well [17–19].

We present the largest single center study of EBUS sampling with NGS. The yield we obtained with protocol adherence in our study (66%) aligns with yields reported in other studies (60–77%) including those by Hagemann et al. [11], Fielding et al. [20], and Rooper et al. [21]. In the study by Hagemann et al., of 381 patients with NSCLC, about 55% of samples were successfully sequenced [11]. When broken down by method of collections, endoscopic yields were found to be 66% (47 of 71 samples). Fielding et al. tested 66 EBUS samples of NSCLC and were able to successfully perform NGS testing in 60.1% (*n* = 40) [20]. Rooper et al. had a 76.6% success rate on 107 EBUS samples [21]. A systematic review and meta-analysis by Zhao et al. in 2022 reported a higher average yield, but the selection criteria for samples in some of the included studies makes it difficult to compare directly with our study [12]. The meta-analysis examined 21 studies comprising 1175 patients and found a pooled proportion of sufficient EBUS samples for NGS of 86.5% but some of these studies pre-selected samples for adequacy before attempting NGS testing [12]. For example, Stoy et al. reports a 95% success rate but samples were selected from a pathology database [22]. Likewise, Turner et al. assessed 315 NSCLC of which 200 were not tested and 115 were attempted for NGS testing [23]. They had an 86% success rate, but NGS testing was performed in-house where initial processing may preserve cells for NGS. Interestingly, 17 of those were pre-determined to be “borderline” for possible adequacy, suggesting that some samples excluded from NGS testing for perceived inadequacy might be able to yield results. Overall, pre-selection, exclusion of “borderline” results, and the use of in-house NGS processing may all contribute to higher NGS yields in these latter mentioned studies compared to ours. The substantial increase in yield we observed when our protocol was adhered to compared to non-adherence, supports its application and widespread use, although further studies are needed to optimize the protocol in hopes of improving yields even further.

The limitations of this study include its retrospective nature and its reliance on potentially incomplete documentation. NGS sequencing was not attempted on 84 samples and may be underpowered to detect size differences in yields. Not testing may be due to Oncology not requesting sampling (either because it was not needed or patient did not consent to testing or to treatment). Furthermore, reasons for deviation from the sampling protocol was not collected but may have included items such as difficult aspiration, bleeding, or poor patient tolerance of the procedure (thus limiting the time allowed for collection), or other clinical decompensation event (such as hypoxia). However, no difference was found in protocol adherence for moderate sedation versus general anesthesia (57.4% vs 35.7%, *p* = 0.27). If patient factors are the limiting agent for protocol adherence, then improved protocols may not result in improved study outcomes or tissue sample yields; however, there were few complications so patient factors are likely not an impactful limitation.

The strengths of this study include its robust sample size spanning a 6-year period. It fills a gap in the literature in regard to NSG yields and prediction factors, an area where prior studies are scarce and with smaller samples sizes.

When choosing biopsy locations for molecular genetics and NGS, proceduralists should consider aiming for larger lesions and obtaining three or more aspirations for NGS. Future studies comparing NSG yields between various sampling protocols, including reflex testing, should be considered to determine the ideal method for collecting and processing tissue samples for genetic sequencing. Reflex testing for NGS with upfront processing of the cell block may reduce material waste from preparation and result in higher yields.

## Data Availability

Data is provided within the manuscript.
